# Left atrial dysfunction can independently predict exercise capacity in patients with chronic heart failure who use beta-blockers

**DOI:** 10.1186/s12872-023-03127-9

**Published:** 2023-03-09

**Authors:** Pengtao Sun, Huan Cen, Sinan Chen, Xiankun Chen, Wei Jiang, Huiying Zhu, Yuexia Liu, Hongmei Liu, Weihui Lu

**Affiliations:** 1 The Second School of Clinical Medicine, Southern Medical University, Guangzhou, 510000 China; 2 The Second Affiliated Hospital of Guangzhou University of Chinese Medicine, Guangzhou, 510000 China; 3 Department of Ultrasonography, Guangdong Provincial Hospital of Chinese Medicine, Guangzhou, 510000 China; 4 State Key Laboratory of Dampness Syndrome of Chinese Medicine, The Second Affiliated Hospital of Guangzhou University of Chinese Medicine, Guangzhou, 510000 China; 5 Key Unit of Methodology in Clinical Research, Guangdong Provincial Hospital of Chinese Medicine, Guangzhou, 510000 China; 6 Health Systems and Policy, Department of Global Public Health, Karolinska Institutet, 17177 Stockholm, Sweden; 7 Department of Cardiology, Guangdong Provincial Hospital of Chinese Medicine, Guangzhou, 510000 China; 8 Department of Ultrasonography, Institute of Ultrasound in Musculoskeletal Sports Medicine, Guangdong Second Provincial General Hospital, Guangzhou, 510000 China

**Keywords:** Left atrial strain, Chronic heart failure, Exercise capacity, Maximal oxygen uptake, Beta-blockers

## Abstract

**Background:**

Beta-blockers are first-line clinical drugs for the treatment of chronic heart failure (CHF). In the guidelines for cardiac rehabilitation, patients with heart failure who do or do not receive beta-blocker therapy have different reference thresholds for maximal oxygen uptake (VO_2max_). It has been reported that left atrial (LA) strain can be used to predict VO_2max_ in patients with heart failure, which can be used to assess exercise capacity. However, most existing studies included patients who did not receive beta-blocker therapy, which could have a heterogeneous influence on the conclusions. For the vast majority of CHF patients receiving beta-blockers, the exact relationship between LA strain parameters and exercise capacity is unclear.

**Methods:**

This cross-sectional study enrolled 73 patients with CHF who received beta-blockers. All patients underwent a thorough resting echocardiogram and a cardiopulmonary exercise test to obtain VO_2max_, which was used to reflect exercise capacity.

**Results:**

LA reservoir strain, LA maximum volume index (LAVI_max_), LA minimum volume index (LAVI_min_) (*P* < 0.0001) and LA booster strain (*P* < 0.01) were all significantly correlated with VO_2max_, and LA conduit strain was significantly correlated with VO_2max_ (*P* < 0.05) after adjusting for sex, age, and body mass index. LA reservoir strain, LAVI_max_, LAVI_min_ (*P* < 0.001), and LA booster strain (*P* < 0.05) were significantly correlated with VO_2max_ after adjusting for left ventricular ejection fraction, the ratio of transmitral E velocity to tissue Doppler mitral annulus e′ velocity (E/e′), and tricuspid annular plane systolic excursion. LA reservoir strain with a cutoff value of 24.9% had a sensitivity of 74% and specificity of 63% for the identification of patients with VO_2max_ < 16 mL/kg/min.

**Conclusion:**

Among CHF patients receiving beta-blocker therapy, resting LA strain is linearly correlated with exercise capacity. LA reservoir strain is a robust independent predictor of reduced exercise capacity among all resting echocardiography parameters.

*Clinical Trial registration*: This study is a part of the Baduanjin-Eight-Silken-Movement with Self-efficacy Building for Patients with Chronic Heart Failure (BESMILE-HF) trial NCT03180320 (ClinicalTrials.gov, registration date: 08/06/2017).

## Key points

- A significant linear correlation exists between resting LA strain and exercise capacity in CHF patients receiving beta-blocker therapy.
- LA reservoir strain is the strongest independent predictor of decreased exercise capacity among all LA strain parameters.
- LA booster strain is also an independent predictor of decreased exercise capacity.

## Introduction

Beta-blockers are first-line clinical drugs for the treatment of chronic heart failure (CHF). They can reduce exercise heart rate, increase exercise fatigue, and reduce maximum oxygen uptake (VO_2max_) by approximately 7–15% [1, 2]. Therefore, in the guidelines for cardiac rehabilitation, patients with heart failure who do or do not receive beta-blocker therapy have different reference thresholds for VO_2max_ [3]. Previous studies have reported that left atrial (LA) reservoir strain, conduit strain and booster strain measured by speckle-tracking echocardiography can predict the VO_2max_ of patients with heart failure, which can be used to reflect exercise capacity [4, 5]. However, to the best of our knowledge, most existing studies have not limited the use of beta-blockers in the enrolled population. Therefore, the enrollment of a small number of patients with heart failure who did not use beta-blockers could lead to heterogeneity and influence study conclusions. In the real world, the majority of patients with heart failure use beta-blockers. No studies have reported the relationship between LA dysfunction and exercise capacity in patients with heart failure who do or do not receive beta-blocker therapy; thus, the results are unclear. Therefore, the main objectives of our study were to investigate the relationship between LA strain and exercise capacity in a CHF population who used beta-blockers.

## Methods

### Setting

This study involved patients from the Outpatient Clinic, Cardiac Rehabilitation Division, Ersha Branch, Guangdong Provincial Hospital of Chinese Medicine.

### Study population

This was a cross-sectional study as well as a part of the Baduanjin-Eight-Silken-Movement with Self-efficacy Building for Patients with Chronic Heart Failure (BESMILE-HF) trial (NCT03180320, ClinicalTrials.gov, registration date: 08/06/2017) [6]. Patients with CHF were prospectively recruited between February 2019 and July 2022 if they fulfilled the following inclusion criteria: (1) ≥ 18 years of age; (2) met the diagnostic criteria for CHF [7]; (3) clinically stable, defined as symptoms/signs that remained generally unchanged for ≥ 1 month; (4) New York Heart Association class II or III; (5) used beta-blockers; and (6) provided informed consent [8].

**Fig. 1:**
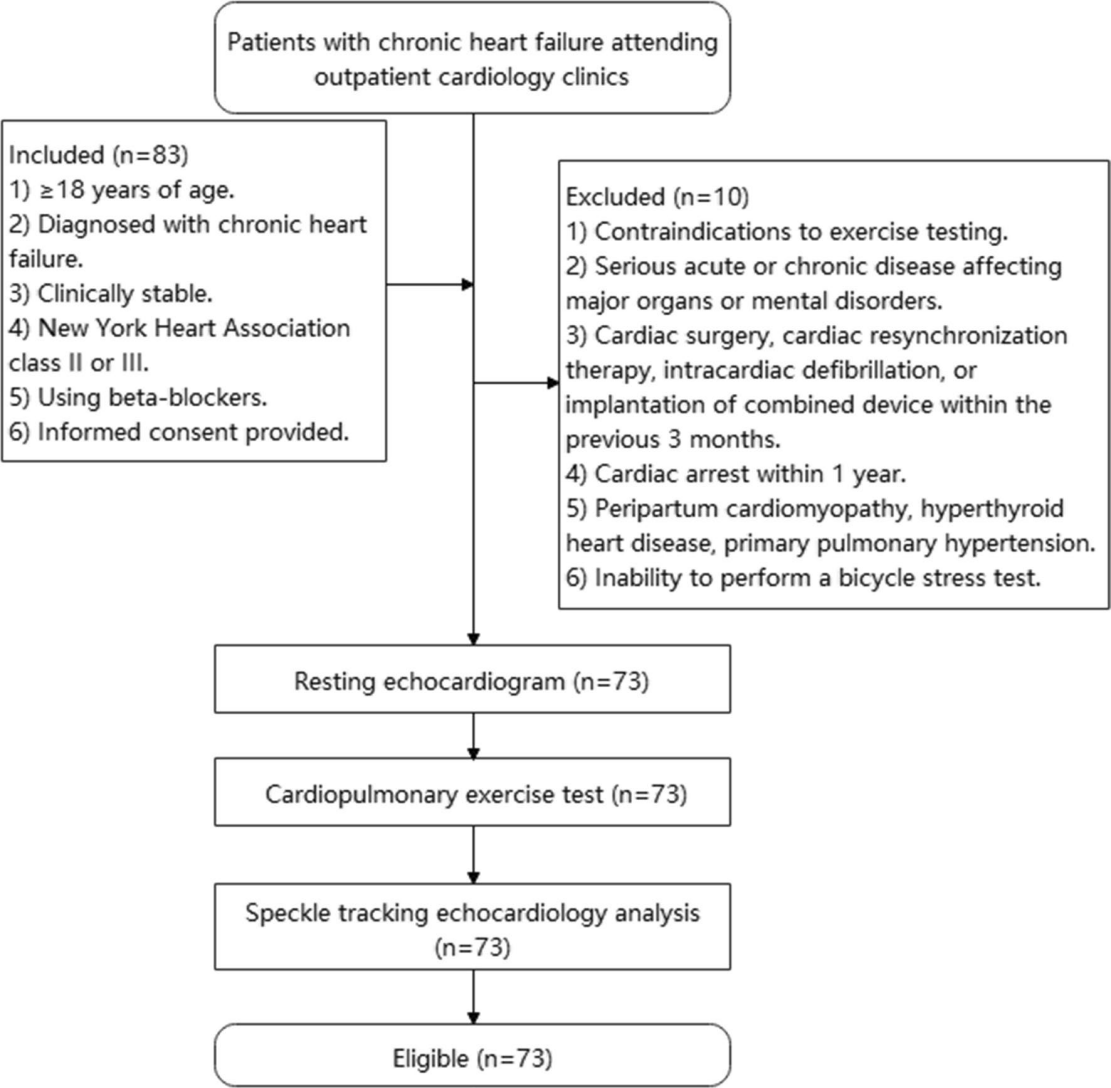
Flow chart of this study

The exclusion criteria were as follows: (1) patients with contraindications for exercise testing, namely, early phase after acute coronary syndrome (up to 6 weeks), life-threatening cardiac arrhythmias, acute heart failure (during the initial period of hemodynamic instability), uncontrolled hypertension (systolic blood pressure > 200 mmHg and/or diastolic blood pressure > 110 mmHg), advanced atrioventricular block, acute myocarditis and pericarditis, moderate to severe aortic valve/mitral stenosis, severe aortic valve/mitral regurgitation, severe hypertrophic obstructive cardiomyopathy, acute systemic illness, or intracardiac thrombus; (2) patients with serious acute or chronic diseases affecting major organs or with mental disorders; (3) patients with a history of cardiac surgery, cardiac resynchronization therapy, intracardiac defibrillation, or implantation of a combined device within the previous 3 months; (4) patients with a history of cardiac arrest within 1 year; (5) patients with a history of peripartum cardiomyopathy, hyperthyroid heart disease, or primary pulmonary hypertension; and (6) patients unable to perform a recumbent bicycle stress test (Fig. 1) [6].

**Fig. 2:**
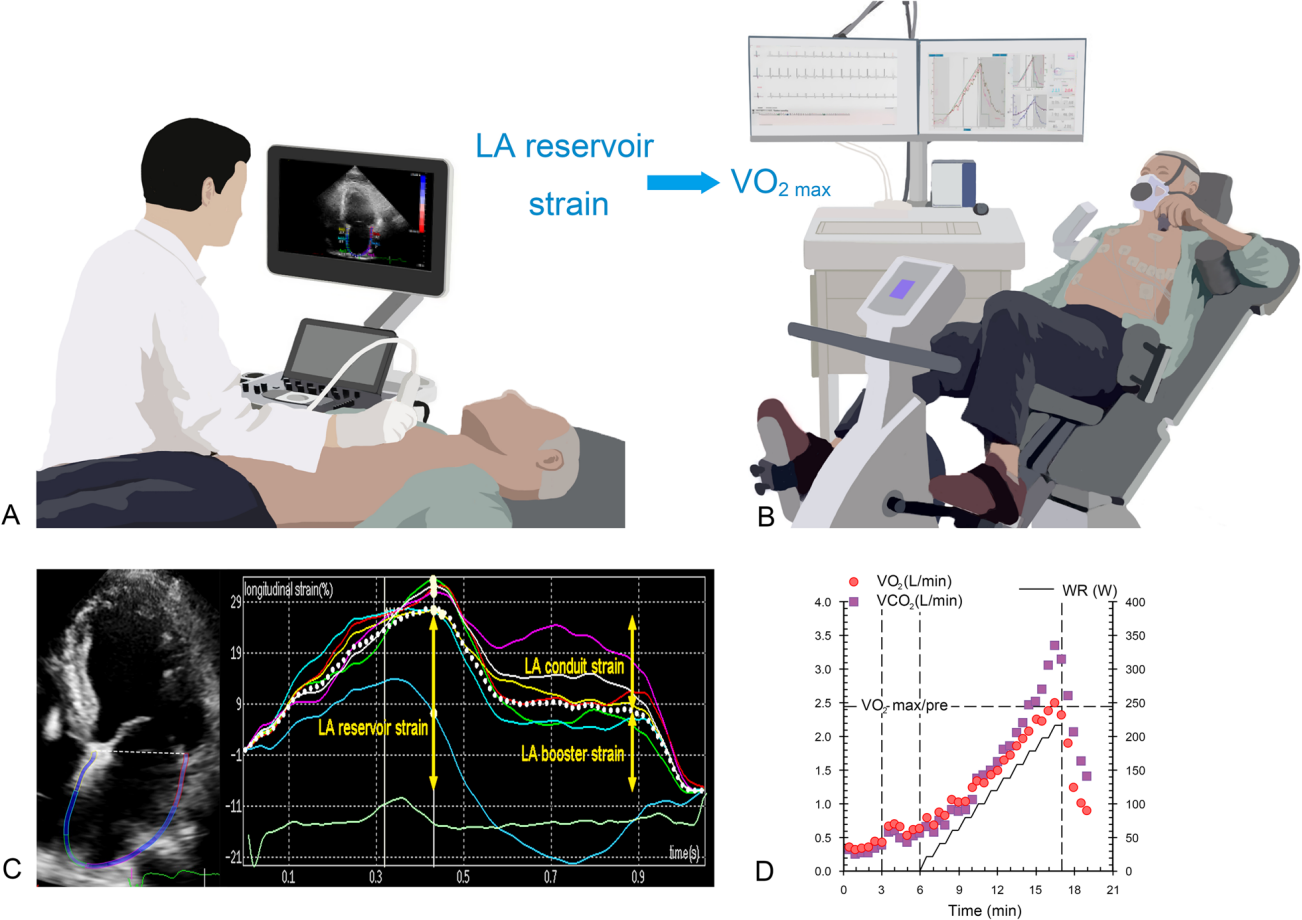
Illustration of speckle-tracking echocardiography examination (**A**) and cardiopulmonary exercise testing (**B**). Strain analysis of the left atrium in the locally enlarged apical four-chamber view and the LA strain curve throughout the cardiac cycle (**C**). The curves of VO_2_ and VCO_2_ with time and work rate, respectively (**D**). LA, left atrial; VO_2_, oxygen uptake; VCO_2_, carbon dioxide uptake; VO_2max/pre_, ratio of maximum to predicted oxygen uptake, WR, work rate

Eligible participants underwent clinical evaluation (including history of cardiac risk factors and medications), height and weight measurements, blood testing, and electrocardiography. They then underwent a cardiopulmonary exercise test (CPET) and transthoracic echocardiography assessment at rest on the same day (Fig. 2A, B). The BESMILE-HF study[6] was approved by the Ethics Committee of the Guangdong Provincial Hospital of Chinese Medicine (Approval No. B2016-202-01). All of the participants provided written informed consent.

### Cardiopulmonary exercise test

The CPET was performed using a standard ramp protocol (initial work rate of 0 W with an increase of 10% per minute of the predicted maximum work rate, which was calculated with a previously described formula[9]). The maximal exercise test was performed on an electronically calibrated recumbent bicycle (ERG 911S; Schiller, Baar, Switzerland). The patients started at 3 min of no-load cycling (0 W) and maintained pedaling with stepwise increases in the workload by 10% of the predicted VO_2max_ every minute until they reached the maximum perceived effort [10]. Finally, the patient was instructed to ride the recumbent bicycle for 3 min without any load. The peak VO_2_ was averaged over 30 s as usually prescribed [3, 11]. Respiratory gas analysis was performed on a breath-by-breath basis with a metabolic cart (PowerCube, Ganshorn, Germany) and LF8 software (CARDIOVIT, CS-200 ergospirometer; Schiller AG, Switzerland). VO_2max_ and the maximum respiratory exchange ratio (RER_max_) were measured during an adequately performed test (Fig. 2B, D).

### Echocardiography

Transthoracic echocardiography was performed with the patients at rest on an EPIQ 7C ultrasound system (Philips Healthcare, Amsterdam, the Netherlands) according to the guidelines of the American Society of Echocardiography (2019) [12].

Additional optimized left ventricular (LV) and LA images were acquired at a frame rate > 50 frames/s. In apical 4-, 3-, and 2-chamber views of 3 continuous cardiac cycles at rest, the early diastolic peak flow velocity of the mitral valve on pulsed-wave Doppler (E), the early diastolic velocity of the septal and lateral wall annulus on tissue Doppler (e′) and the ratio of E to e′ (E/e′) were calculated. Three-dimensional full-volume images at rest were acquired to calculate the LV volume, stroke volume and ejection fraction. All images were stored digitally. The volume and strain analyses were performed offline using commercially available software (QLab 10.8.0; Philips Healthcare). The LA maximum and minimum volume index (LAVI_max_ and LAVI_min_, respectively) and the LA total empty fraction (LATEF) were evaluated from the apical 4- and 2-chamber views by the 2-dimensional quantitative speckle-tracking method [13].

### Speckle-tracking echocardiography

All images were analyzed by a single investigator (S.C.) who was blinded to the participants’ characteristics and exercise performance. For the LA strain, the LA endocardium was manually traced at the end systole stage of LV, and the software automatically tracked the myocardium throughout the cardiac cycle on electrocardiography using R-to-R gating. Figure 2C shows the LA strain curve throughout the cardiac cycle. The region of interest was adjusted to the smallest LA wall thickness for tracking. The LA strain is the average value measured in the 4- and 2-apical chamber views. In the reservoir phase, as the left atrium filled and stretched, there was a positive atrial strain that peaked in systole at the end of LA filling and before the opening of the mitral valve; this was the LA reservoir strain, which was defined as the difference between the nadir and the peak of the strain curve. Elevated LAVI was defined as LAVI_max_ ≥ 34 mL/m^2^, while reduced LA reservoir strain was defined as LA reservoir strain < 23% [4, 14]. Subsequent passive LA emptying with the opening of the mitral valve was observed, with negative deflection of the strain curve until a plateau was reached; this was the LA conduit strain, which was defined as the difference between the peak of the strain curve and the onset of atrial contraction following the P wave. Then, a second negative deflection in the strain curve was observed corresponding to atrial systole, which represented LA active contraction. The LA booster strain was defined as the difference between the onset of atrial contraction and the nadir of the strain curve [13, 14] (Fig. 2C).

The LV endocardium was traced at end systole in the 4-, 3-, and 2- apical views; the region of interest was selected with software and was adjusted to accommodate the thickness of the LV myocardium. The LV global longitudinal strain (LVGLS) was measured as the average strain of 17 segments.

### Statistical analysis

This was an observational cross-sectional study. The predictor variables were LA strains. Potential covariates or confounders were LA volume index, LV function, right ventricular function, age, sex, body mass index (BMI), biochemical index, etc. The outcome variable was VO_2max_.

The independent sample t test was used to compare data with a normal distribution between groups. Data that did not conform to a normal distribution were compared using the rank sum test. Linear regression was performed using VO_2max_ as the dependent variable. Univariate regression was used to evaluate the independent contribution of LA strain and LA volume to VO_2max_. Multivariate linear regression was used to combine clinical and LV function correction models.

Receiver operating characteristic (ROC) curve analysis was used to evaluate the area under the curve (AUC) for LA strain to predict impaired VO_2max_, which was defined as VO_2max_ < 16 mL/min/kg. This reference value of VO_2max_ was based on the Weber classification [15, 16]. DeLong's test was used to compare the AUC values.

Statistical analysis was performed using STATA 17.0 (STATA Corp, College Station, TX, USA) and MedCalc 20.1.0 (MedCalc Software Ltd., Ostend, Belgium). All tests were two-tailed, and *P* < 0.05 was considered statistically significant. Continuous variables are expressed as the mean ± standard deviation, and categorical variables are expressed as numbers and percentages.

## Results

### Patient characteristics

Of the 83 CHF patients treated with beta-blockers included in the study, 10 were excluded due to medical histories or clinical conditions (Fig. 1). Therefore, the final sample included 73 patients (mean age of 61 ± 10 years; 78% men). In the overall population, 35 patients (48%) had a VO_2max_ of < 16 mL/kg/min, and 38 patients had a VO_2max_ (52%) of ≥ 16 mL/kg/min. In general, patients with a reduced VO_2max_ were older, were more likely to be female, had a higher NT-proBNP level, had a slightly lower hemoglobin level, and had worse renal function. There was no significant difference in comorbidities or combined medications between the two groups (Table 1).

**Table 1:** Baseline clinical characteristics of the patients with chronic heart failure who underwent cardiopulmonary exercise testing (n = 73)

Variables	Overall population (n = 73)	VO_2max_ ≥ 16 mL/kg/min (n = 38)	VO_2max_ < 16 mL/kg/min (n = 35)	*P* value for comparison	*P* value for correlation with linear VO_2max_
*Demographics*					
Age, years	61 ± 10	57 ± 10	65 ± 9	0.001**	< 0.0001***
Male	57 (78%)	34 (90%)	23 (66%)	0.014*	0.004**
Height, cm	166 ± 7	168 ± 7	164 ± 7	0.001**	0.022*
Weight, kg	68 ± 11	69 ± 12	67 ± 10	0.569	0.530
BMI, kg/m^2^	25 ± 4	24 ± 3	25 ± 4	0.249	0.414
BSA, m^2^	1.8 ± 0.2	1.8 ± 0.2	1.7 ± 0.2	0.270	0.303
*Comorbidities and etiology*					
Coronary heart disease	43 (59%)	26 (68%)	17 (49%)	0.085	0.349
Myocardial infarction	20 (27%)	12 (32%)	8 (23%)	0.404	0.595
Dilated cardiomyopathy	19 (26%)	9 (24%)	10 (29%)	0.634	0.491
Hypertrophic cardiomyopathy	2 (3%)	1 (3%)	1 (3%)	0.953	0.485
NVM	2 (3%)	1 (3%)	1 (3%)	0.953	0.939
Moderate mitral regurgitation	16 (22%)	6 (16%)	10 (26%)	0.187	0.171
Diabetes	30 (41%)	16 (42%)	14 (40%)	0.860	0.999
Hypertension	32 (44%)	15 (39%)	17 (49%)	0.434	0.062
Hyperlipidemia	26 (36%)	13 (34%)	13 (37%)	0.794	0.752
Hyperuricemia	26 (36%)	11 (29%)	15 (43%)	0.215	0.036*
CKD (compensation stage)	7 (10%)	3 (8%)	4 (11%)	0.608	0.291
COPD	4 (5%)	3 (8%)	1 (3%)	0.360	0.670
*Laboratory variables*					
NT-proBNP, pg/mL	892 ± 1417	516 ± 664	1301 ± 1855	< 0.0001***	0.004**
Hemoglobin, g/L	145 ± 15	149 ± 12	139 ± 16	0.003**	0.003**
Creatinine, mg/dL	95 ± 23	98 ± 23	91 ± 24	0.695	0.371
Sodium, mEq/L	139 ± 6	139 ± 7	140 ± 3	0.636	0.055
GFR, mL/min/1.73 m^2^	73 ± 17	76 ± 18	69 ± 16	0.082	0.044*
Glucose, mmol/L	7 ± 2	6 ± 2	7 ± 2	0.947	0.720
Triglycerides, mmol/L	1.5 ± 1.4	1.7 ± 1.8	1.4 ± 0.6	0.800	0.140
Cholesterol, mmol/L	4.2 ± 1.5	4.3 ± 1.8	4.1 ± 1.1	0.774	0.322
HDL, mmol/L	1.21 ± 0.28	1.21 ± 0.25	1.2 ± 0.3	0.995	0.682
nHDL, mmol/L	3.0 ± 1.5	3.1 ± 1.8	2.8 ± 1.1	0.770	0.265
LDL, mmol/L	2.4 ± 0.9	2.4 ± 0.8	2.4 ± 1.0	0.943	0.748
*Oral drugs*					
Inhibitors of RAS	48 (66%)	28 (74%)	20 (57%)	0.137	0.078
Spironolactone	47 (64%)	19 (50%)	28 (80%)	0.007**	0.003**
ARNI	15 (21%)	7 (18%)	8 (23%)	0.639	0.487
CCB	7 (10%)	2 (5%)	5 (14%)	0.204	0.411
P2Y12 receptor antagonist	29 (40%)	17 (45%)	12 (34%)	0.362	0.510
Statin	53 (73%)	26 (68%)	27 (77%)	0.404	0.390
Trimetazidine	7 (10%)	3 (8%)	4 (11%)	0.608	0.308
Aspirin	21 (29%)	10 (26%)	11 (31%)	0.630	0.878
Warfarin	10 (14%)	4 (11%)	6 (17%)	0.411	0.445

* *P* < 0.05, ** *P* < 0.01, *** *P* < 0.001

Data are presented as the mean ± standard deviation or the n (%). BMI, body mass index; BSA, body surface area; CKD, chronic kidney disease; COPD, chronic obstructive pulmonary disease; GFR, glomerular filtration rate; HDL, high-density lipoprotein. HR, heart rate; LDL, low-density lipoprotein; NT-proBNP, N-terminal pro-brain natriuretic peptide; nHDL, nonhigh-density lipoprotein; NVM, noncompaction of ventricular myocardium; RAS renin-angiotensin system; ARNI, angiotensin receptor neprilysin inhibitor; CCB, calcium channel blocker

### Echocardiography parameters and VO_2max_

Univariate linear regression showed that E/e′ (*P* < 0.0001), E, left ventricular end systolic volume index (LVESVI) (above *P* < 0.01), left ventricular end diastolic volume index (LVEDVI), and left ventricular ejection fraction (LVEF) (all *P* < 0.05) were significantly correlated with VO_2max_ (Table 2). The LV systolic function parameters LVGLS and left ventricular stroke volume index (LVSVI) and the right ventricular systolic function parameter tricuspid annular plane systolic excursion (TAPSE) were not significantly correlated with VO_2max_.

**Table 2:** Resting echocardiography parameters and cardiopulmonary exercise test parameters of the patients with chronic heart failure (n = 73)

Variables	Total (n = 73)	VO_2max_ ≥ 16 mL/kg/min (n = 38)	VO_2max_ < 16 mL/kg/min (n = 35)	*P* value for comparison	*P* value for correlation with VO_2max_ in linear regression analysis
*Left ventricular diastolic variables*					
E, cm/s	82 ± 31	75 ± 25	90 ± 36	0.106	0.005**
e′ _ave_, cm/s	6.5 ± 2.2	6.8 ± 2.1	6.2 ± 2.4	0.174	0.277
E/e′	13.6 ± 6.0	11.6 ± 3.9	15.7 ± 7.1	0.006**	< 0.0001***
*Left ventricular systolic variables*					
LVEDVI, ml/m^2^	80 ± 43	69 ± 20	93 ± 57	0.059	0.015*
LVESVI, ml/m^2^	48 ± 35	38.30 ± 15	58 ± 47	0.079	0.008**
LVSVI, ml/m^2^	32 ± 10	31 ± 7	34 ± 13	0.212	0.280
LVEF, %	44 ± 10	46 ± 8	42 ± 12	0.182	0.020*
LVEF ≥ 50%, n(%)	25 (34%)	15 (39%)	10 (29%)	0.143	–
LVEF = 41–49%, n (%)	21 (29%)	13 (34%)	8 (23%)		
LVEF ≤ 40%, n (%)	27 (37%)	10 (26%)	17 (49%)		
LVGLS, %	− 13.0 ± 3.5	− 13.3 ± 3.1	− 12.7 ± 4.0	0.431	0.191
TAPSE, cm	2.0 ± 0.4	2.0 ± 0.3	2.0 ± 0.5	0.760	0.300
*Left atrial variables*					
LA reservoir strain, %	24.3 ± 9.2	27.5 ± 9.7	20.9 ± 7.3	0.003**	< 0.0001***
LA conduit strain, %	− 12.9 ± 6.9	− 14.1 ± 7.2	− 11.6 ± 6.4	0.100	0.019*
LA booster strain, %	− 11.4 ± 6.5	− 13.5 ± 6.1	− 9.3 ± 6.3	0.005**	0.001**
LAVI_max_, ml/m ^2^	31.1 ± 18.4	23.6 ± 9.7	39.3 ± 21.9	0.0002***	< 0.0001***
LAVI_min_, ml/m ^2^	17.8 ± 14.8	11.8 ± 6.6	24.3 ± 18.2	0.0001***	< 0.0001***
LATEF, %	47 ± 14	51 ± 14	42 ± 12	0.004**	< 0.0001***
*CPET variables*					
RER_max_	1.15 ± 0.08	1.17 ± 0.07	1.13 ± 0.08	0.032*	0.027*
Load_max_, W	89 ± 32	110 ± 26	67 ± 20	< 0.0001***	< 0.0001***
VE/VCO_2_ slope	32 ± 6	29 ± 4	36 ± 6	< 0.0001***	< 0.0001***
VO_2max/pre_, %	63 ± 14	70 ± 11	55 ± 12	< 0.0001***	< 0.0001***
VO_2max_, mL/kg/min	17 ± 4	20 ± 3	13 ± 2	< 0.0001***	–
O_2_ pulse_max_, mL/beat	9 ± 3	11 ± 2	8 ± 2	< 0.0001***	< 0.0001***
O_2_ pulse_max_/_pre_,%	78 ± 18	85 ± 14	70 ± 20	0.0006***	< 0.0001***
HR _max_, bpm	126 ± 25	131 ± 19	121 ± 30	0.068	0.011*

*P < 0.05, ** *P* < 0.01, *** *P* < 0.001

Data are presented as the mean ± standard deviation. E, early diastolic peak flow velocity of the mitral valve; e′_ave_, average early diastolic velocity of mitral annulus; E/e′, ratio of transmitral E velocity to tissue Doppler mitral annulus e′ velocity; LATEF, left atrial total empty fraction; LA, left atrial; LAVI_max_, maximum left atrial volume index; LAVI_min_, minimum left atrial volume index; LVEDVI, left ventricular end diastolic volume index; LVEF, left ventricular ejection fraction; LVESVI, left ventricular end systolic volume index; LVGLS, left ventricular global longitudinal strain; RER_max_, maximum respiratory exchange ratio; Load_max_, maximum load; TAPSE, tricuspid annular plane systolic excursion; VE/VCO_2_, minute ventilation to carbon dioxide production; VO_2max_, maximum oxygen uptake; VO_2max/pre_, ratio of maximum to predicted oxygen uptake; HR_max_, maximum heart rate; O_2_ pulse_max_, maximum oxygen consumption per pulse; O_2_ pulse _max/pre_, ratio of maximum to predicted oxygen consumption per pulse

### LA function and VO_2max_

**Fig. 3:**
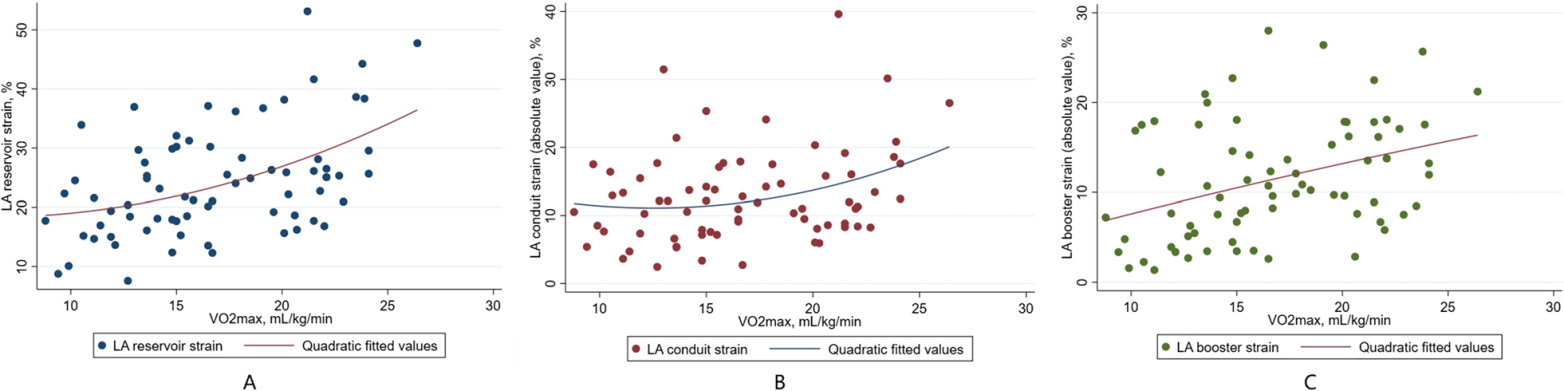
Linear correlations between LA reservoir strain (**A**), conduit strain (**B**), booster strain (**C**) and VO_2max_ values in the overall study population. LA, left atrial; VO_2max_, maximum oxygen uptake

**Fig. 4:**
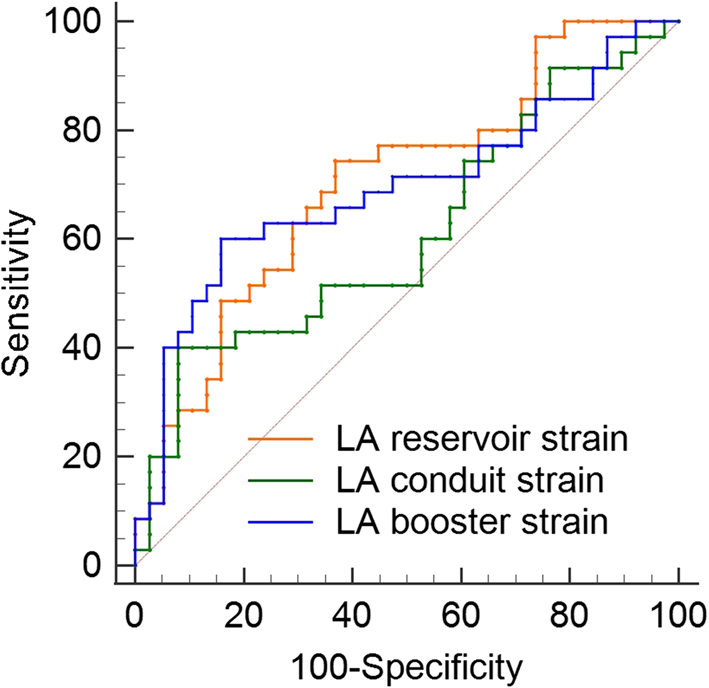
Receiver operating characteristic curves of LA strain in the univariable regression model for predicting VO_2max_ < 16 mL/kg/min. LA, left atrial

This study explored LA function. In the overall population, 22 patients (30%) had an elevated LAVI (≥ 34 mL/m^2^), and 36 patients (49%) showed a reduced LA reservoir strain (< 23%) [4, 14]. When VO_2max_ decreased, LAVI increased, and the absolute value of LA strain decreased (Fig. 3). An LA reservoir strain of < 24.9% predicted a VO_2max_ of < 16 mL/kg/min with high specificity and moderate sensitivity in this study (Table 3, Fig. 4).

**Table 3:** Receiver operating characteristic curve analysis of the parameters with the greatest area under the curve

Variable	Cutoff value	Sensitivity (%)	Specificity (%)	AUC	95%CI	*P*
LA reservoir strain	24.9%	74.29	63.16	0.70	0.59 to 0.81	< 0.001***
LA conduit strain	− 7.9%	40.00	92.11	0.61	0.49 to 0.72	0.097
LA booster strain	− 8.0%	60.00	84.21	0.69	0.57 to 0.80	0.003**
LVEF	38.9%	45.71	81.58	0.59	0.47 to 0.71	0.189
TAPSE	18 mm	51.52	74.19	0.56	0.43 to 0.69	0.396

**P < 0.01; *** *P* < 0.001

AUC, area under the curve; CI, confidence interval; LA, left atrial; LVEF, left ventricular ejection fraction; TAPSE, tricuspid annular plane systolic excursion

Considering the results of univariate regression, clinical significance, and reducing collinearity of variables to avoid overfitting, the predictor variables of this study were LA reservoir strain, LA conduit strain, LA booster strain, LAVI_max_ and LAVI_min_. Potential covariates or confounders were LVEF, E/e', TAPSE, age, sex, BMI, NT-proBNP level and hemoglobin level, which were selected based on the positive results of the univariate regression and in combination with clinical physiology and pathology[4] (Tables 1, 2). The outcome variable was VO_2max_. Therefore, we used three multivariate regression models to examine the independent effect of LA strain. The results showed that LA reservoir strain, LAVI_max_, LAVI_min_ (*P* < 0.001), and LA booster strain (*P* < 0.05) were significantly correlated with VO_2max_ in the 3 models (Table 4).

**Table 4:** Association between LA parameters, clinical or other echocardiographic variables, and VO_2max_ (mL/kg/min) in the univariate and multivariate linear regression analyses: ***P*** value, coefficient (95% confidence interval)

	LA reservoir strain	LA conduit strain	LA booster strain	LAVI_max_	LAVI_min_
Univariate	< 0.0001;0.23 (0.13 to 0.33)	0.019; 0.18 (0.03 to 0.32)	0.001; 0.25 (0.10 to 0.41)	< 0.0001; − 0.13 ( − 0.17 to − 0.08)	< 0.0001; − 0.16 ( − 0.22 to − 0.10)
Model 1	< 0.0001;0.19 (0.09 to 0.28)	0.004; 0.19 (0.06 to 0.32)	0.032; 0.16 (0.010 to 0.31)	< 0.0001; − 0.10 ( − 0.15 to − 0.05)	< 0.0001; − 0.13 ( − 0.19 to − 0.07)
Model 2	0.001; 0.19 (0.08 to 0.29)	0.140; − 0.143 ( − 0.28 to − 0.00)	0.015; − 0.18 ( − 0.33 to − 0.04)	< 0.0001; − 0.10 ( − 0.16 to − 0.05)	< 0.0001; − 0.13 ( − 0.21 to − 0.06)
Model 3	0.003; 0.18 (0.07 to 0.29)	0.114; − 0.12 ( − 0.28 to 0.03)	0.021; − 0.19 ( − 0.35 to − 0.03)	0.002; − 0.10 ( − 0.15 to − 0.04)	0.003; − 0.11 ( − 0.19 to − 0.04)

Model 1: adjusted for age, sex, and BMI. Model 2: adjusted for NT-proBNP, hemoglobin and GFR. Model 3: adjusted for left ventricular ejection fraction, E/e' and TAPSE

LA, left atrial; LAVI_max_, maximum left atrial volume index; LAVI_min_, minimum left atrial volume index; BMI, body mass index; NT-proBNP, N-terminal pro-brain natriuretic peptide; GFR, glomerular filtration rate; E/e′, ratio of transmitral E velocity to tissue Doppler mitral annulus e′ velocity; TAPSE, tricuspid annular plane systolic excursion

**Fig. 5:**
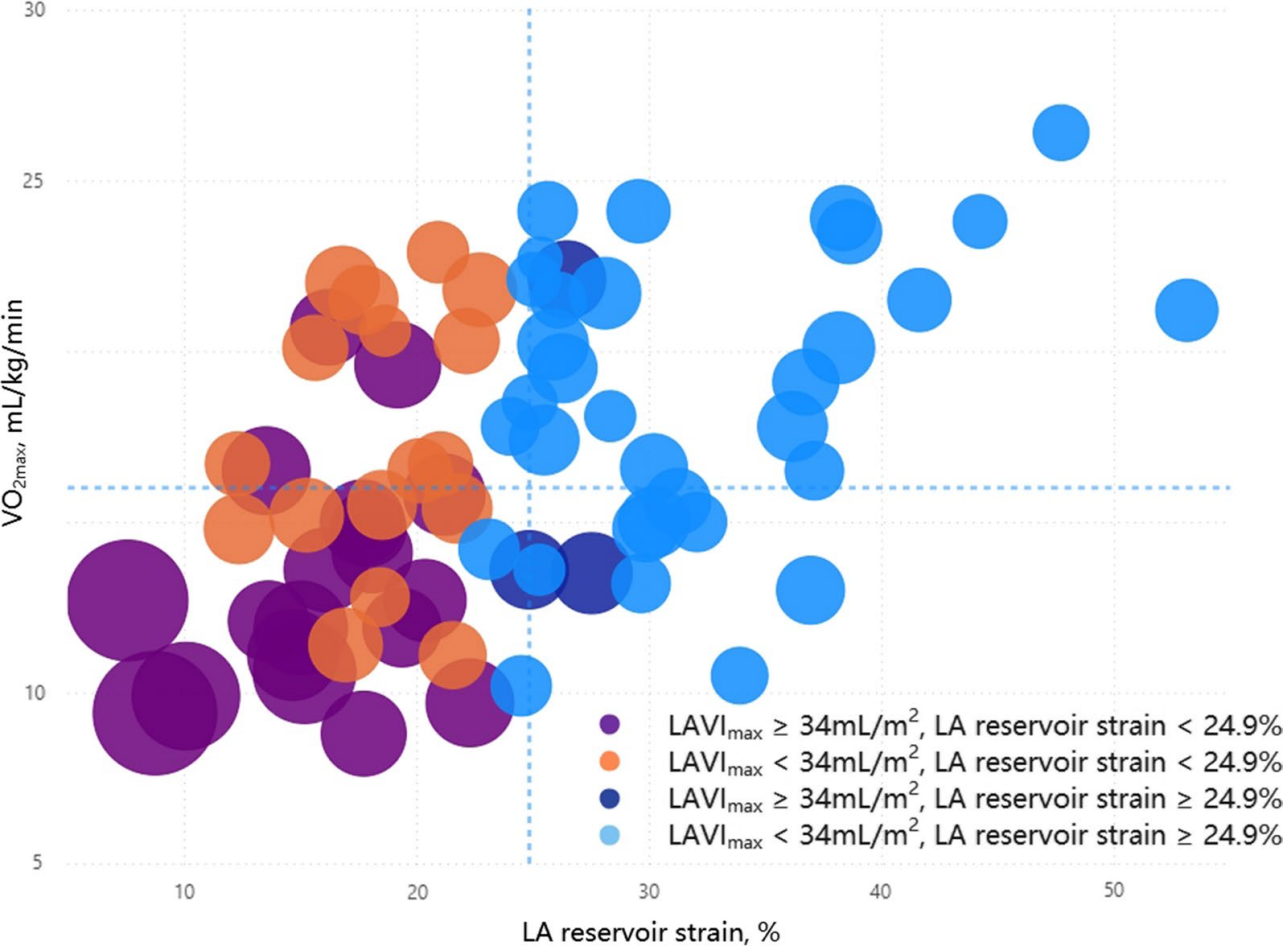
Bubble chart of LA reservoir strain < or ≥ 24.9% and normal/dilated LAVI_max_ in patients with chronic heart failure. The size of the bubble: LAVI_max_. The x-axis of constant: LA reservoir strain = 24.9%. The y-axis of constant: VO_2max_ = 16 mL/kg/min. LA, left atrial; LAVI_max_, maximum left atrial volume index; VO_2max_, maximum oxygen uptake

In our study, the lower the LA reservoir strain was, the lower VO_2max_ was, and the larger LAVI_max_ was (Fig. 5). The VO_2max_ of most patients with increased LAVI_max_ and decreased LA reservoir strain was less than 16 mL/kg/min (Fig. 5, lower left quadrant); the VO_2max_ of most patients with normal LAVI_max_ and normal LA reservoir strain was greater than 16 mL/kg/min (Fig. 5, upper right quadrant).

## Discussion

Beta-blockers are first-line drugs for the treatment of heart failure, and they are administered to the majority of heart failure patients [7]. In cardiac rehabilitation guidelines, patients with heart failure who receive beta-blockers and those who do not receive beta-blockers have different reference thresholds for VO_2max_ [3]. To avoid a heterogeneous effect of beta-blockers on assessing the association between LA dysfunction and reduced exercise capacity in patients with CHF, our study assessed only patients with CHF who received beta-blockers. To the best of our knowledge, our study is the first to confirm that in CHF patients receiving beta-blocker therapy (1) a linear correlation exists between resting LA strain and exercise capacity; (2) among all LA strain parameters, LA reservoir strain is the strongest independent predictor for detecting decreased exercise capacity and for identifying patients with VO_2max_ < 16 mL/kg/min; notably, LA reservoir strain has high sensitivity and moderate specificity, with a critical value of 24.9%; and 3) LA booster strain was also an independent predictor of decreased exercise capacity.

### Effect of beta-blockers on maximal oxygen uptake

As widely used drugs for the treatment and management of patients with CHF [7], beta-blockers have a significant impact on the regulation of the heart and peripheral blood vessels and can reduce the mortality and hospitalization rate in CHF patients [17]. When the β-receptors of the heart are blocked, the exercise heart rate is 20–30% lower than that of patients not taking beta-blockers, but the stroke volume increases by 5–23% to maintain sufficient cardiac output [18]. Due to the increase in stroke volume, myocardial work efficiency increases, myocardial oxygen consumption increases, and VO_2max_ slightly decreases [1, 19]. In addition, β-receptor blockade causes peripheral vasodilation, pulmonary bronchoconstriction, and increased muscle and liver glycogenolysis; furthermore, this phenomenon affects the oxygen transport and work efficiency of skeletal muscles [20]. These conditions exist with both selective and nonselective beta-blockers.

### Exercise capacity and maximal oxygen uptake in CHF patients

Cardiopulmonary dysfunction and skeletal muscle atrophy are the main causes of reduced exercise capacity in patients with heart failure [21]. With the progression of CHF, cardiac systolic and diastolic dysfunction leads to decreased exercise capacity, which is caused by impaired cardiac output, hemodynamic changes, and myocardial remodeling [22]. In addition, oxidative stress and proinflammatory conditions lead to muscle atrophy associated with a decrease in CHF physical function [23]. The exercise capacity of the included patients, represented by VO_2max_, was generally reduced, mainly due to heart-related cardiopulmonary function impairment.

### LA dysfunction and reduced exercise capacity in CHF patients

Our study of patients with CHF who received beta-blockers showed that, compared with other echocardiographic parameters, LA reservoir strain was the strongest biomarker for predicting the exercise capacity of these patients. LA reservoir strain with a cutoff value of 24.9% had high sensitivity and moderate specificity in the detection of VO_2max_ < 16 mL/kg/min in all subjects. LA booster strain with a cutoff value of -8.0% had moderate sensitivity and high specificity in the detection of VO_2max_ < 16 mL/kg/min in all subjects. LA strain has been considered a useful echocardiographic marker in a series of patients with heart diseases and as an independent predictor of all-cause mortality [24, 25]. In terms of exercise capacity evaluation, previous heart failure studies that did not limit beta-blockers only found that LA reservoir strain was associated with VO_2max_ and VE/VCO_2_slope [26]. LA reservoir strain can specifically reflect the pathophysiological mechanism of LA, such as LA myocardial remodeling and dysfunction [27], which can increase hemodynamic stress and pulmonary vascular resistance, resulting in a decrease in VO_2max _[28].

A recent clinical study involving myocardial biopsy demonstrated that both LA reservoir strain and LA volume were predictors of LA fibrosis [29]. LAVI is a marker of LV systolic and diastolic dysfunction[30] and has been shown to be associated with exercise tolerance in HF patients [31]. However, an increased LA volume is unlikely to completely reflect the complex phenomenon of LA remodeling. Therefore, the introduction of LA strain can be used to more accurately evaluate the physiological and pathological LA changes in CHF patients; additionally, the predictive ability of LA reservoir strain is slightly better than that of LA volume [4, 29]. In our study, LA strain and exercise capacity were closely and independently correlated, and LA reservoir strain had the strongest predictive ability among the LA strain parameters in patients with CHF.

After adjusting for LVEF, E/e′, and TAPSE, LA reservoir strain still had a good ability to predict exercise capacity (P = 0.003). Considering that the interaction between atrial phasic function and ventricular mechanics regulates cardiovascular function, our findings are not surprising. Because the increase in preload led to more severe LA dysfunction [32], LA dysfunction in turn aggravated heart failure [33].

Advanced age, BMI and sex are important factors in exercise capacity [34]. In a healthy population, LA reservoir strain showed a decreasing trend with increasing age [35]. In our study, LA reservoir strain was linearly correlated with VO_2max_ after adjusting for age, sex, and BMI. These results suggest that the value of LA reservoir strain for predicting exercise capacity in patients with CHF is not affected by those clinical factors.

Our study showed that LA booster strain was also an independent predictor of VO_2max_. In previous studies, it was unclear whether LA booster strain could independently predict VO_2max_ [4, 36, 37]. The previously reported contradictory findings may have been related to the limitation of LA booster strain measurement accuracy due to the inclusion of some patients with atrial fibrillation. In our study, only one patient had a history of paroxysmal atrial fibrillation. Due to the inhibitory effect of beta-blockers on atrial fibrillation, this patient did not develop atrial fibrillation during the study period. Relatively accurate LA conduit strain and LA booster strain values were obtained. LA booster strain represents the LA mechanical changes during the late LV diastole [13]. After active LA contraction, the pressure gradient from the left atrium to the left ventricle increases, and the left ventricle is further filled. Inoue’s [36] study found that there was a strong correlation between LA booster strain and LA reservoir strain (r = 0.81, *P* < 0.001). The decrease in LA pressure after atrial contraction increases the pressure gradient from the pulmonary vein to the LA, thereby increasing LA filling [36]. Therefore, the active booster function of the left atrium is one of the determinants of LA reservoir strain. Unlu’s [38] study proved that LA booster strain is the only LA strain parameter that can independently reflect the intrinsic LA function and is not affected by changes in blood volume because in the late LV diastolic phase (LA systolic phase), the participation of the left atrium in blood volume is relatively small, and it is almost unrelated to volume load. After the use of beta-blockers, the heart rate is reduced, which is manifested as prolonged diastolic time. As a result, the LA contraction time is relatively increased. Thus, LA booster strain in CHF patients using beta-blockers is more attributable to LA function.

Most LV filling is completed in the early LV diastole, and LA conduit strain is more susceptible to the effect of LV diastolic function than LA booster strain. Therefore, in our study, we corrected the representative parameter of diastolic function, which is also the early diastolic E/e′. Consequently, LA conduit strain was no longer an independent predictor of VO_2max_.

## Limitations

Our study had strict inclusion criteria; for example, only patients with stable New York Heart Association classification II or III CHF were included. The sample size was moderate but adequate because the VO_2max_ of nearly half of the subjects was decreased. Second, the peak diastolic flow velocity of the mitral valve (A) or the average late-diastolic velocity of the mitral annulus (a′_ave_) for patients with short-term or other cardiac diseases was not reached in 23 patients (32%) in this study. Therefore, A and a'_ave_ were not included in the univariate regression. Third, severe valvular regurgitation could be a manifestation or cause of CHF and affect LA function. To avoid these confounding factors, our study excluded patients with severe valvular regurgitation and only included patients with mild or moderate valvular regurgitation. Fourth, although there have been an increasing number of studies on LA strain, the current software package for measuring LA strain that was used to evaluate LV strain has inherent limitations. Fifth, the Jones formula we used to calculate the predicted VO_2max_ might overestimate the predicted VO_2max_ values. Finally, this study only included patients who took beta-blockers, and further studies are needed to compare patients who do and do not take beta-blockers.

## Conclusion

This study demonstrated that in patients with CHF who received beta-blockers, LA reservoir strain is a robust independent predictor of reduced exercise capacity among resting echocardiographic parameters. LA booster strain is independently correlated with VO_2max_. An LA reservoir strain of 24.9% can be used to identify patients with VO_2max_ < 16 mL/kg/min with high specificity and moderate sensitivity. LA strain is a potentially useful indicator for evaluating the efficacy of cardiac rehabilitation.

## Data Availability

The datasets used and/or analyzed during the current study are available from the corresponding author on reasonable request.

## Abbreviations

BMI: Body mass index
CHF: Chronic heart failure
CI: Confidence interval
E: Early diastolic peak flow velocity of the mitral valve
e′_ave_: Average early diastolic velocity of mitral annular
E/e′: Ratio of transmitral E velocity to tissue Doppler mitral annular e’_ave_ velocity
LA: Left atrial
LATEF: Left atrial total empty fraction
LAVI_max_: Maximum left atrial volume index
LAVI_min_: Minimum left atrial volume index
LV: Left ventricular
LVEDVI: Left ventricular end diastolic volume index
LVESVI: Left ventricular end systolic volume index
LVEF: Left ventricular ejection fraction
LVGLS: Left ventricular global longitudinal strain
RER_max_: Maximum respiratory exchange rate
TAPSE: Tricuspid annular plane systolic excursion
VO_2max_: Maximum oxygen uptake

## References

[1] Mitchell BL, Davison K, Parfitt G (2019). Physiological and perceived exertion responses during exercise: effect of β-blockade. Med Sci Sports Exerc.

[2] Dodd S, Powers S, O'Malley N (1988). Effects of beta-adrenergic blockade on ventilation and gas exchange during incremental exercise. Aviat Space Environ Med.

[3] Corrà U, Agostoni PG, Anker SD (2018). Role of cardiopulmonary exercise testing in clinical stratification in heart failure. A position paper from the committee on exercise physiology and training of the heart failure association of the European Society of Cardiology. Eur J Heart Fail.

[4] Maffeis C, Rossi A, Cannata L (2022). Left atrial strain predicts exercise capacity in heart failure independently of left ventricular ejection fraction. ESC Heart Failure.

[5] Pugliese NR, De Biase N, Conte L (2021). Cardiac reserve and exercise capacity: insights from combined cardiopulmonary and exercise echocardiography stress testing. J Am Soc Echocardiogr.

[6] Chen X, Jiang W, Lin X (2018). Effect of an exercise-based cardiac rehabilitation program "Baduanjin Eight-Silken-Movements with self-efficacy building" for heart failure (BESMILE-HF study): study protocol for a randomized controlled trial. Trials.

[7] Heidenreich PA, Bozkurt B, Aguilar D (2022). 2022 AHA/ACC/HFSA guideline for the management of heart failure: executive summary: a report of the American College of Cardiology/American Heart Association Joint Committee on Clinical Practice Guidelines. Circulation.

[8] Thomas L, Muraru D, Popescu BA (2020). Evaluation of left atrial size and function: relevance for clinical practice. J Am Soc Echocardiogr.

[9] Jones NL, Makrides L, Hitchcock C (1985). Normal standards for an incremental progressive cycle ergometer test. Am Rev Respir Dis.

[10] Myers J, Bellin D (2000). Ramp exercise protocols for clinical and cardiopulmonary exercise testing. Sports Med.

[11] Pugliese NR, De Biase N, Balletti A (2022). Characterization of hemodynamic and metabolic abnormalities in the heart failure spectrum: the role of combined cardiopulmonary and exercise echocardiography stress test. Minerva Cardiol Angiol.

[12] Mitchell C, Rahko PS, Blauwet LA (2019). Guidelines for performing a comprehensive transthoracic echocardiographic examination in adults: recommendations from the American Society of Echocardiography. J Am Soc Echocardiogr.

[13] Badano LP, Kolias TJ, Muraru D (2018). Standardization of left atrial, right ventricular, and right atrial deformation imaging using two-dimensional speckle tracking echocardiography: a consensus document of the EACVI/ASE/industry task force to standardize deformation imaging. Eur Heart J Cardiovasc Imaging.

[14] Pathan F, D'Elia N, Nolan MT (2017). Normal ranges of left atrial strain by speckle-tracking echocardiography: a systematic review and meta-analysis. J Am Soc Echocardiogr.

[15] Guazzi M, Borlaug B, Metra M (2021). Revisiting and implementing the weber and ventilatory functional classifications in heart failure by cardiopulmonary imaging phenotyping. J Am Heart Assoc.

[16] Smarż K, Jaxa-Chamiec T, Chwyczko T (2019). Cardiopulmonary exercise testing in adult cardiology: expert opinion of the working group of cardiac rehabilitation and exercise physiology of the polish cardiac society. Kardiol Pol.

[17] Nielsen PB, Larsen TB, Gorst-Rasmussen A (2016). β-Blockers in atrial fibrillation patients with or without heart failure: association with mortality in a nationwide cohort study. Circ Heart Fail.

[18] Head A (1999). Exercise metabolism and beta-blocker therapy. An update. Sports Med.

[19] Palau P, Seller J, Domínguez E (2021). Effect of β-blocker withdrawal on functional capacity in heart failure and preserved ejection fraction. J Am Coll Cardiol.

[20] Kaiser P (1984). Physical performance and muscle metabolism during beta-adrenergic blockade in man. Acta Physiol Scand Suppl.

[21] Adachi H (2017). Cardiopulmonary exercise test: the most powerful tool to detect hidden pathophysiology. Int Heart J.

[22] Smarż K, Jaxa-Chamiec T, Chwyczko T (2019). Cardiopulmonary exercise testing in adult cardiology expert opinion of the working group of cardiac rehabilitation and exercise physiology of the polish cardiac society. Kardiol Polska.

[23] Howden EJ, Bigaran A, Beaudry R (2019). Exercise as a diagnostic and therapeutic tool for the prevention of cardiovascular dysfunction in breast cancer patients. Eur J Prev Cardiol.

[24] Jia F, Chen A, Zhang D (2022). Prognostic value of left trial strain in heart failure: a systematic review and meta-analysis. Front Cardiovasc Med.

[25] Weerts J, Barandiarán Aizpurua A, Henkens M (2021). The prognostic impact of mechanical atrial dysfunction and atrial fibrillation in heart failure with preserved ejection fraction. Eur Heart J Cardiovasc Imaging.

[26] Leite L, Mendes SL, Baptista R (2017). Left atrial mechanics strongly predict functional capacity assessed by cardiopulmonary exercise testing in subjects without structural heart disease. Int J Cardiovasc Imaging.

[27] Benfari G, Mandoli G, Magne J (2022). Left atrial strain determinants across heart failure stages; insight from MASCOT registry. Eur Heart J Cardiovasc Imaging.

[28] Bhatt A, Flink L, Lu DY (2021). Exercise physiology of the left atrium: quantity and timing of contribution to cardiac output. Am J Physiol Heart Circ Physiol.

[29] Lisi M, Mandoli GE, Cameli M (2022). Left atrial strain by speckle tracking predicts atrial fibrosis in patients undergoing heart transplantation. Eur Heart J Cardiovasc Imaging.

[30] Gehlken C, Screever EM, Suthahar N (2021). Left atrial volume and left ventricular mass indices in heart failure with preserved and reduced ejection fraction. ESC Heart Fail.

[31] Rossi A, Cicoira M, Bonapace S (2007). Left atrial volume provides independent and incremental information compared with exercise tolerance parameters in patients with heart failure and left ventricular systolic dysfunction. Heart.

[32] Bastos MB, Burkhoff D, Maly J (2020). Invasive left ventricle pressure-volume analysis: overview and practical clinical implications. Eur Heart J.

[33] Sun M, Xing Y, Guo Y (2022). Left atrial reservoir strain is an outstanding predictor of adverse cardiovascular outcomes in patients undergoing maintenance hemodialysis: assessment via three-dimensional speckle tracking echocardiography. Clin Cardiol.

[34] Roibal Pravio J, Barge Caballero E, Barbeito Caamaño C (2021). Determinants of maximal oxygen uptake in patients with heart failure. ESC Heart Fail.

[35] Stefani LD, Trivedi SJ, Ferkh A (2021). Changes in left atrial phasic strain and mechanical dispersion: effects of age and gender. Echocardiography.

[36] Inoue K, Khan FH, Remme EW (2021). Determinants of left atrial reservoir and pump strain and use of atrial strain for evaluation of left ventricular filling pressure. Eur Heart J Cardiovasc Imaging.

[37] Gan GCH, Bhat A, Chen HHL (2021). Left atrial reservoir strain by speckle tracking echocardiography: association with exercise capacity in chronic kidney disease. J Am Heart Assoc.

[38] Ünlü S, Yamak BA, Sezenöz B (2021). Left atrial contractile longitudinal strain determines intrinsic left atrial function regardless of load status and left ventricular deformation. Int J Cardiovasc Imaging.

